# Malignant Carotid Body Tumor in a Young Female Patient: Case Report with Review of Literature

**DOI:** 10.7759/cureus.100324

**Published:** 2025-12-29

**Authors:** Mohammad Aadil, Azam Haseen, Aamir Mohammad, Syed Shamayal Rabbani

**Affiliations:** 1 Surgery, Jawaharlal Nehru Medical College, Aligarh Muslim University, Aligarh, IND; 2 Cardiothoracic Surgery, Jawaharlal Nehru Medical College, Aligarh Muslim University, Aligarh, IND

**Keywords:** carotid body tumour, case report, malignant carotid body tumour, malignant paraganglioma, neck mass, shamblin type iii

## Abstract

Carotid body tumors (CBTs) are rare paragangliomas of the head and neck region. They are typically benign and slow-growing but malignant transformation can occur. Early diagnosis and complete surgical excision of the tumors play a vital role in management.

We present the case of an 18-year-old female patient from a non-mountainous region, who presented to us with a gradually progressive, painless swelling that had persisted for two-and-a-half years. The swelling was firm, non-tender, pulsatile, and located on the right side in the anterior triangle of neck with a thrill on palpation. Diagnosis was established by imaging and complete surgical excision was performed. Histopathological examination confirmed the diagnosis of CBT with regional lymph node involvement, establishing the diagnosis of malignancy. The rarity of malignancy and its occurrence in a young female patient make this case noteworthy.

## Introduction

Carotid body tumors (CBTs) are rare neuroendocrine tumors originating from the paraganglionic tissue at the site of bifurcation of the common carotid artery (CCA) [1]. They occur most commonly between the third and sixth decades of life and present more commonly in women [2]. CBTs typically present as slow-growing, painless, pulsatile lateral neck swellings, mobile laterally but less mobile vertically, i.e., in cranio-caudal direction, also known as the Fontaine's sign [3]. Progressive symptoms of dysphagia, hoarseness of voice, odynophagia and cranial nerve deficit (IX-XII) can also appear. Most paragangliomas are solitary, but familial syndromes with multiple paragangliomas and pheochromocytomas are also recognized [2]. Malignant CBTs are uncommon and defined by the presence of spread to regional lymph nodes or the presence of distant metastasis rather than histologic atypia [4]. Metastasis to regional lymph nodes can occur in 5-10% of cases, but distant metastasis is exceedingly rare, with incidence of roughly 1-2% of cases [5]. Early diagnosis using imaging and complete surgical excision remains the mainstay of treatment.

## Case presentation

**Figure 1:**
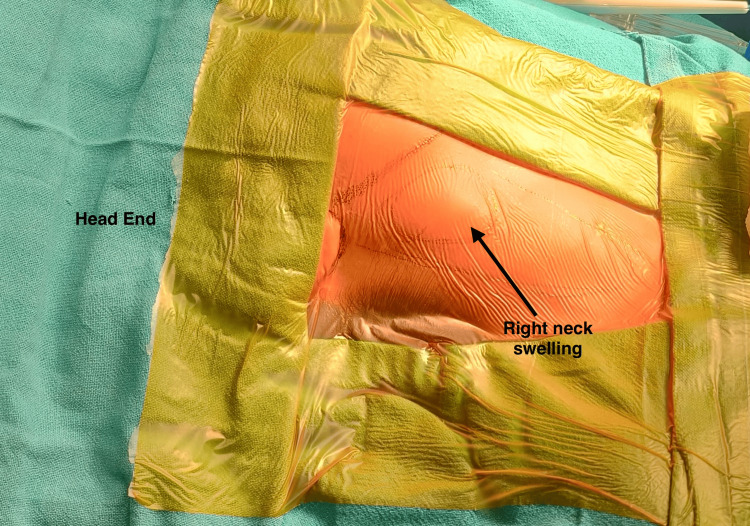
Showing the swelling in the right anterior triangle of neck.

An 18-year-old female patient from a low-altitude region presented to our outpatient department with a history of a gradually progressive, painless swelling on the right side of the neck over the course of two-and-a-half years. There was no history of pain, syncope, dizziness, postural hypotension, dysphagia, hoarseness, or any cranial nerve deficit and no family history of similar complaints. On examination, a 4 × 3 cm firm, pulsatile mass with a thrill was palpable on the right side in the anterior triangle of the neck near the area of carotid bifurcation, anterior to the sternocleidomastoid muscle (Figure 1). It was mobile horizontally but not vertically (positive Fontaine’s sign). No bruit was audible on auscultation and all cranial nerves were intact on examination. There was no palpable lymphadenopathy.

**Figure 2:**
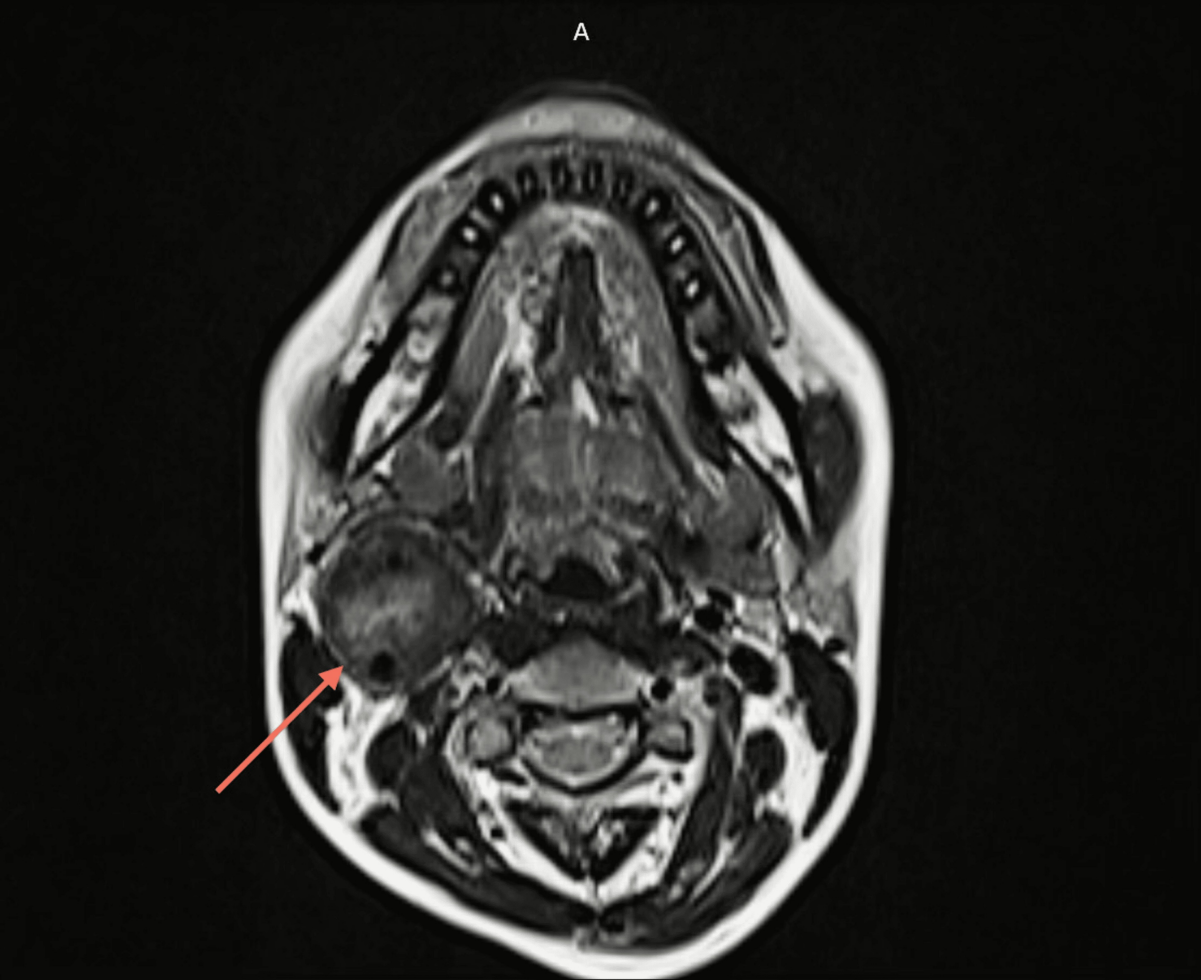
T1-weighted axial section of the magnetic resonance angiography of the head and neck region. The arrow depicts the tumor, with the characteristic salt-and-pepper appearance, on the right side.

**Figure 3:**
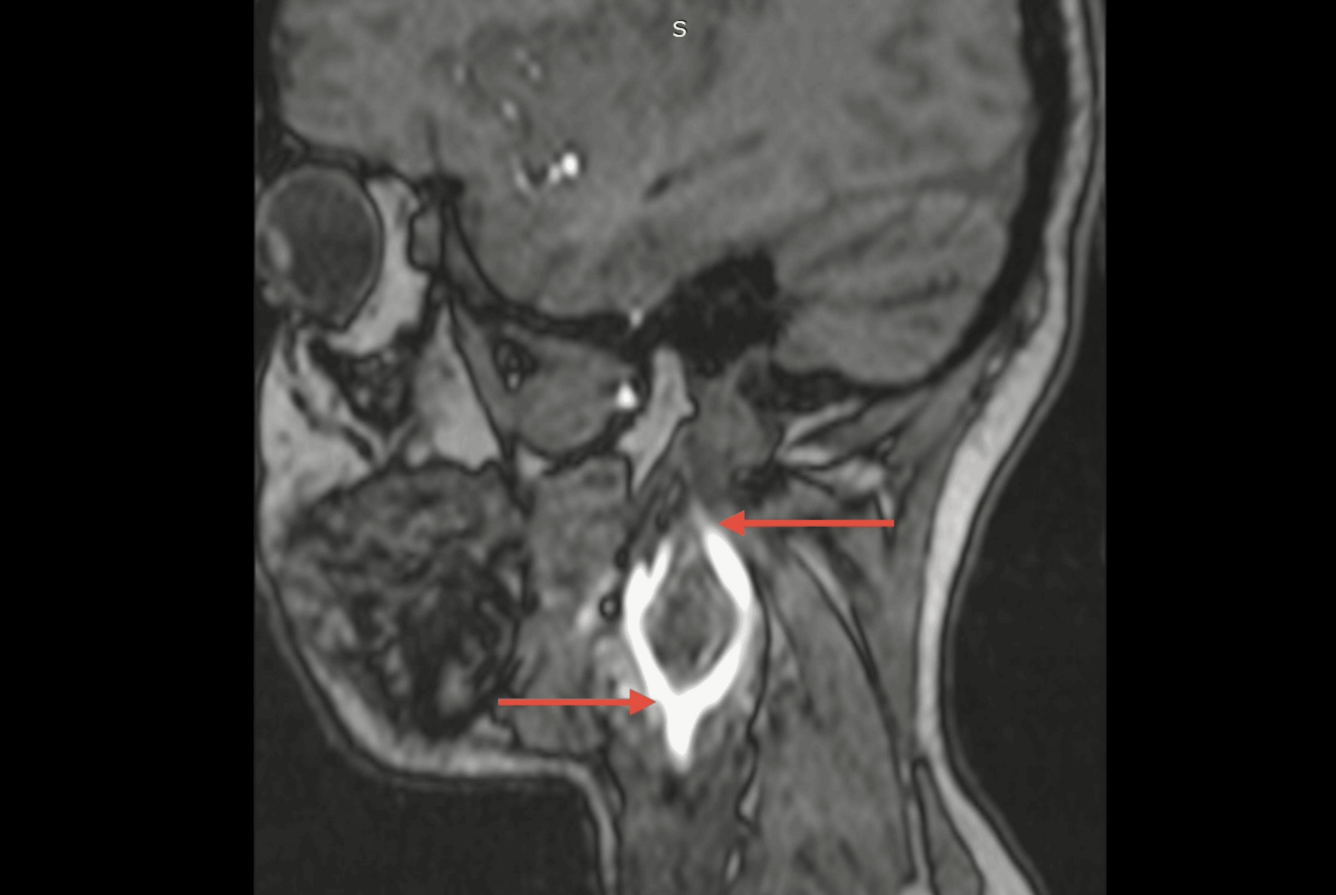
Sagittal section of the magnetic resonance angiography of the head and neck region. The arrows depict splaying of the right ICA and ECA. ECA: External carotid artery; ICA: Internal carotid artery

**Figure 4:**
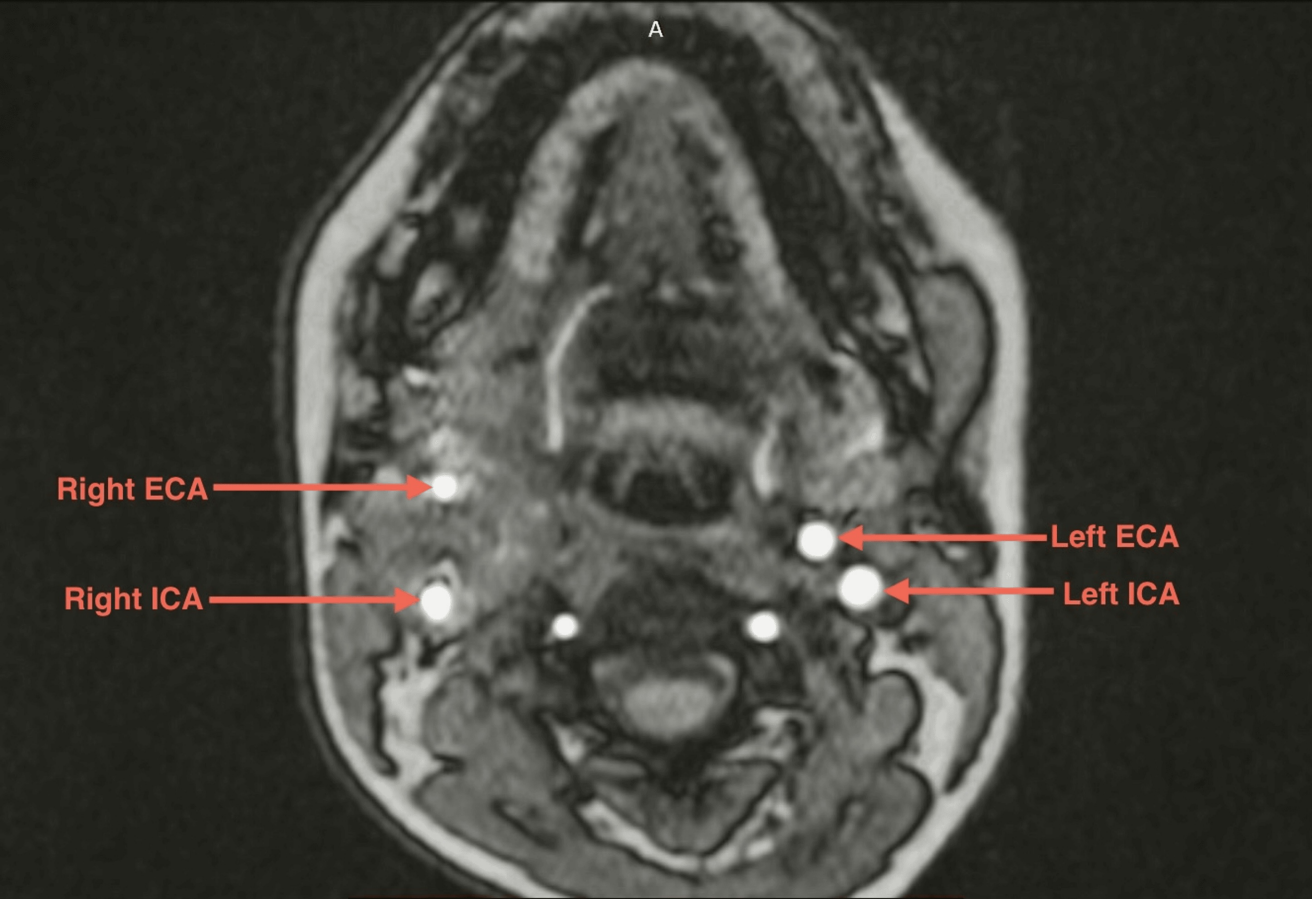
T2-weighted axial section of the magnetic resonance angiography of the head and neck region showing bilateral carotid vessels. Note the difference in the diameter of the right and left vessels on angiogram. ECA: External carotid artery; ICA: Internal carotid artery

Colour doppler was done, which was suggestive of a well-defined hypoechoic vascular soft-tissue lesion at the right carotid bifurcation causing splaying of the internal and external carotid arteries (paraganglionoma). It was followed by magnetic resonance angiography of the head and neck region, which revealed a well-defined soft tissue lesion in right carotid space of size 29 x 29 x 44 mm (anteroposterior x transverse x cranio-caudal) encasing right carotid bifurcation and proximal internal carotid artery (ICA) and external carotid artery (ECA) and causing splaying of the right ICA and ECA with luminal narrowing and reduced flow in right ECA, all of which was suggestive of CBT. Involvement around common carotid from carotid bifurcation ~12.5 mm, around ECA ~21.8 mm, and around ICA ~21 mm, showing the complete involvement of common carotid, ICA and ECA at carotid bifurcation (Shamblin group III) (Figures 2, 3, 4). Tumor was also in close proximity to the cranial nerves (IX-XII) and the planes between them could not be ascertained on imaging.

**Figure 5:**
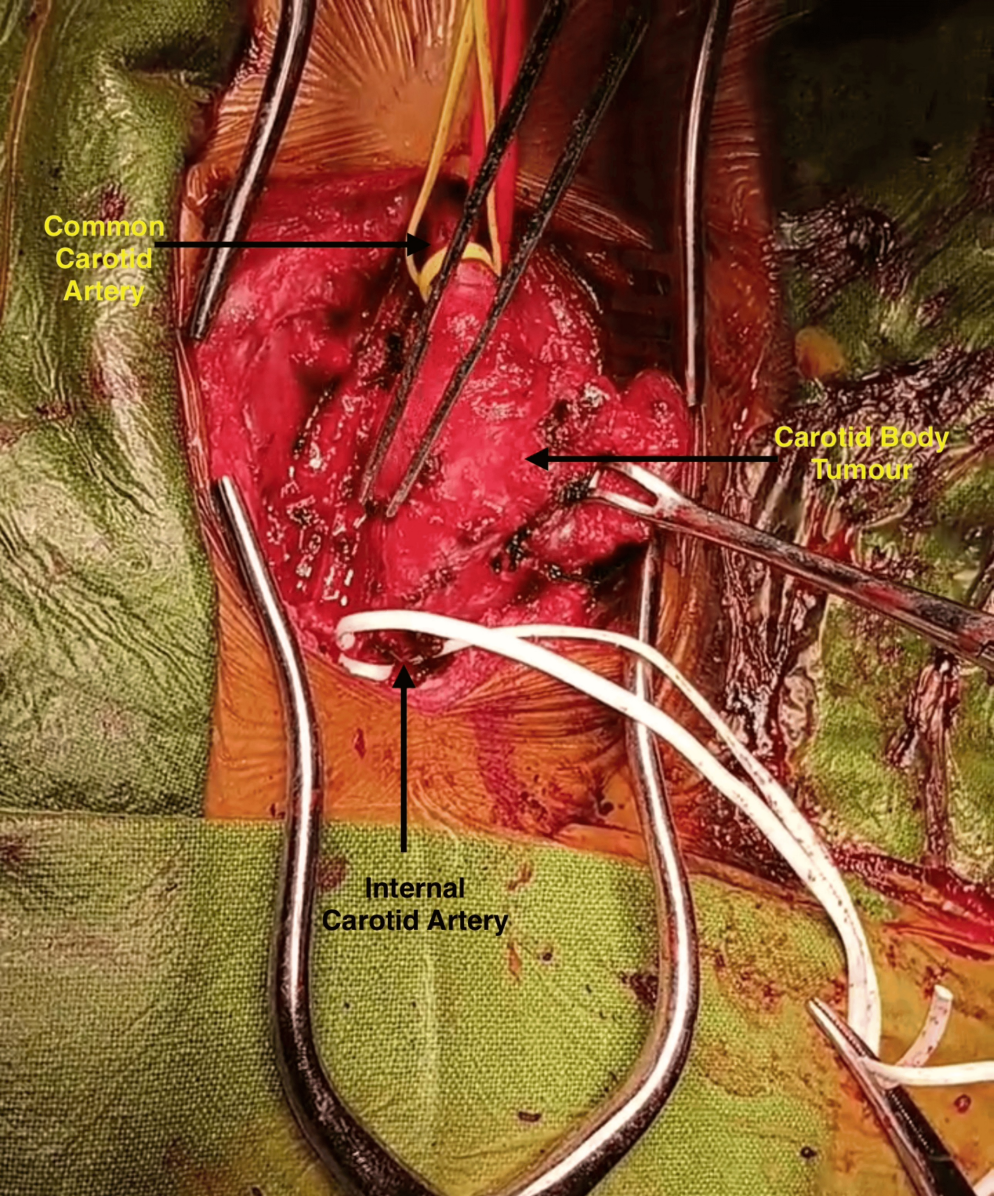
Intraoperative image showing the CBT completely encasing the carotid vessels CBT: Carotid body tumor

**Figure 6:**
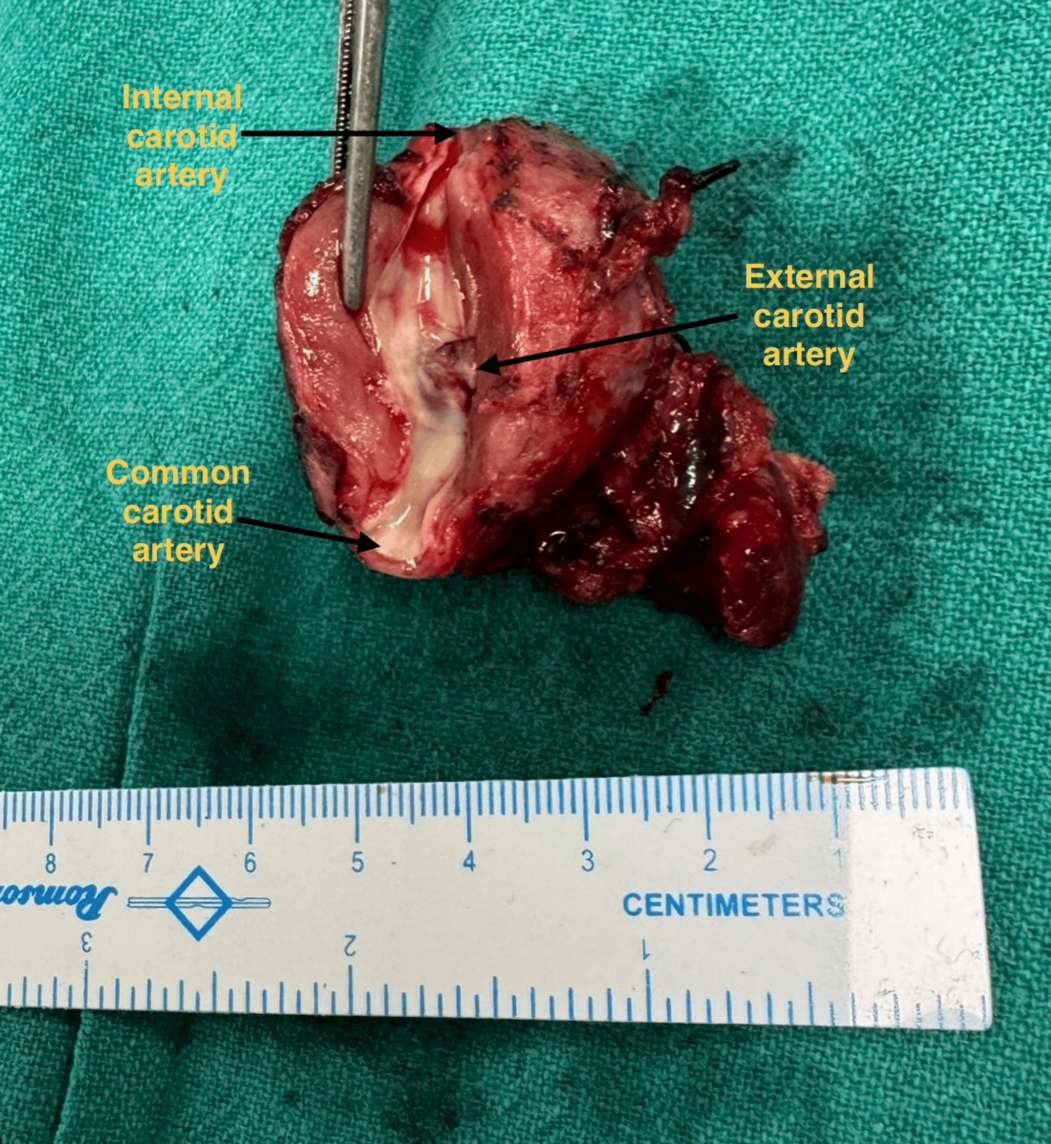
Resected specimen of the tumor showing the carotid vessels. Note the narrowed ostia of the ECA. ECA: External carotid artery

The patient underwent complete excision of the tumor through a standard cervical approach under general anesthesia. Intraoperatively, the tumor was found to completely encase the right common carotid, ECA and ICA with no distinct fat planes (Figure 5). Ostia of the ECA was narrowed with reduced flow while that of ICA was normal with normal flow (Figure 6). Careful subadventitial dissection was done with temporary vascular control as the flow to the circle of willis was complete through the circulation from the opposite side. The tumor was also found to completely invade the cranial nerves (IX-XII); vagus nerve was isolated from the tumor on the right side but other nerves could not be isolated. The tumour was excised in total with repair of right ICA with end-to-end 6 mm polytetrafluoroethylene (PTFE) interposition graft between right CCA and ICA. ECA was ligated as it was very small and thinned out with reduced flow and a romovac suction drain was placed which was removed on third postoperative day.

The patient developed complaint of deviation of angle of mouth to left side and hoarseness of voice postoperatively, for which otorhinolaryngologist consultation was done, and the patient was started on steroids. She was discharged on the fifth postoperative day The patient was followed up in the outpatient department (OPD) one week and four weeks after discharge, showing improvement in her symptoms.

**Figure 7:**
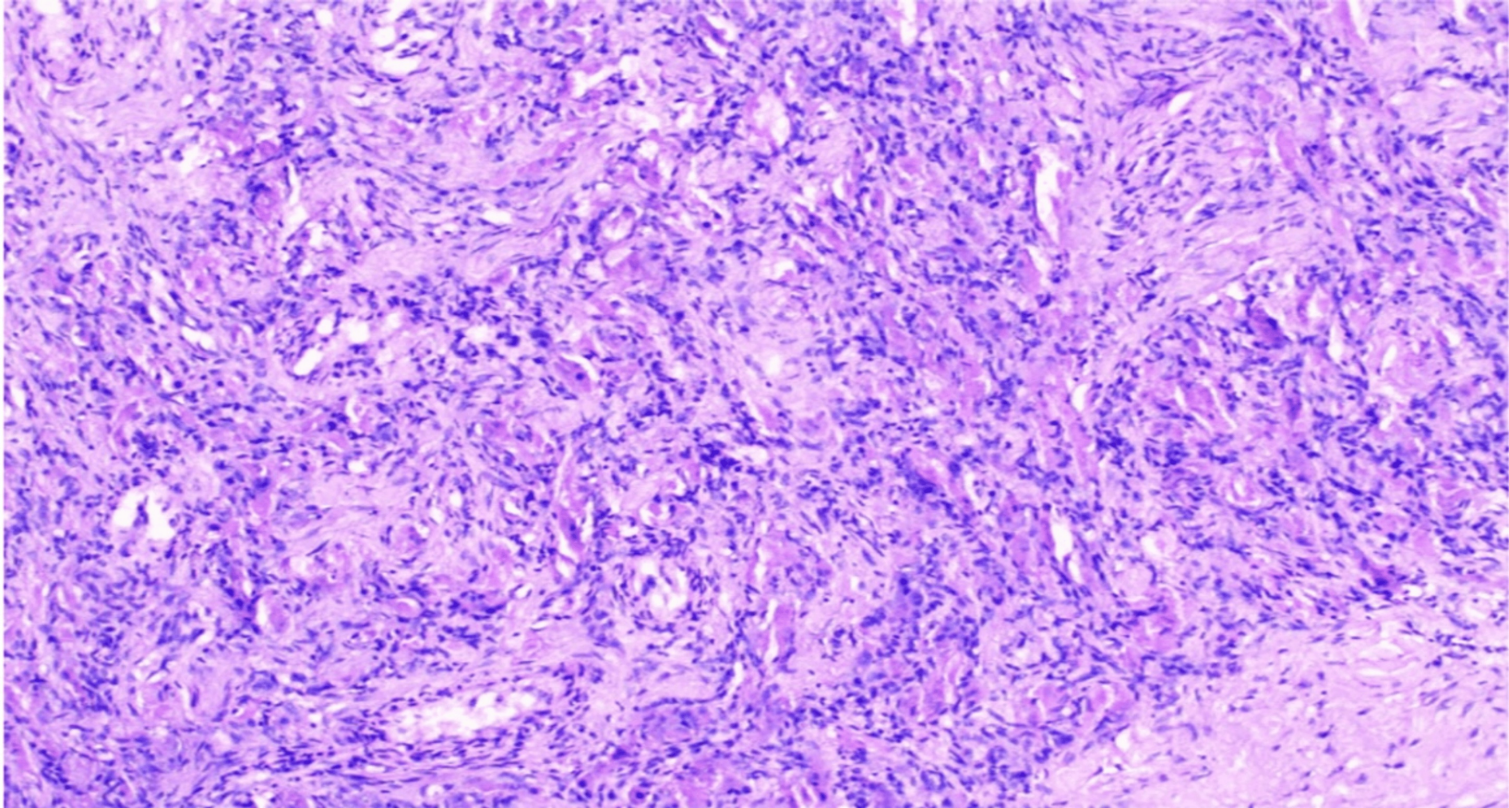
Histopathology of the resected mass showing the characteristic zellballen pattern

Histopathologic examination of the excised mass revealed a "zellballen pattern," consisting of nests, sheets, and trabeculae of ovoid to epithelioid cells with moderate to abundant eosinophilic cytoplasm embedded within fibrotic stroma. Mitotic figures were sparse or absent. Regional lymph nodes were involved, with local infiltration into the surrounding neurovascular structures, suggesting a malignant paraganglioma (malignant CBT) with aggressive behavior (Figure 7). Immunohistochemistry markers were positive for chromogranin and synaptophysin. Genetic screening was advised to the patient but could not be done as it was not available within our institute and due to financial issues.

Consultation was done with radiotherapy department, and adjuvant radiotherapy was recommended to reduce the risk of recurrence. Patient received 60 Gy of radiation in 30 sections using external beam radiotherapy to the right side of neck. Magnetic resonance angiography was done at the three-month follow-up post radiotherapy, and no recurrence was seen.

## Discussion

CBTs, also known as paragangliomas of the carotid body, are rare neoplasms derived from the paraganglionic tissue of neural crest origin located at the bifurcation of the carotid vessels [1]. They account for approximately 0.03% of all tumors and less than 0.5% of head and neck tumors. Although CBTs are typically benign and slow-growing, malignant transformation can occur in 5-10% of cases and is defined by the presence of spread to regional lymph nodes or to distant sites (most common being lungs) [4]. Most CBTs occur sporadically, but up to 30-40% can be familial, associated with germline mutations in the succinate dehydrogenase (SDH) gene complex (SDHB, SDHD, and SDHC) as well as MDH2 and ATRX [6,7]. Other syndromes associated are neurofibromatosis type 1 and von Hippel-Lindau disease [8]. Individuals with hereditary paraganglionomas have early onset of tumors and a higher frequency of bilateral and multiple tumors. Mutations, particularly in SDHB, are associated with higher malignant potential and metastatic risk [9]. The tumor typically affects adults in the third to sixth decades, with a female preponderance (female:male ratio ~3:1) [2]. However, occurrence in adolescence, as in our case, is distinctly unusual.

CBTs are more commonly found in people living at high-altitude regions. As long-term exposure to high altitude can result in chronic hypoxia, which can cause progressive changes in the carotid body, which in turn can affect mechanisms such as cellular energy metabolism and hypoxia inducible factor-1α (HIF-1α). These changes can result in morphological and biochemical alterations in the carotid body, including increased cell size, increased levels of catecholamine, change in cellular appearance, and others [10]. Its diagnosis is by imaging. Magnetic resonance angiography demonstrates the classic 'salt-and-pepper' appearance, and Lyre’s sign is seen on CT angiography [9]. Functional imaging such as 68Ga-DOTATATE positron emission tomography (PET)/CT, octreotide scan, meta-iodobenzylguanidine (MIBG) scanning aids in detecting multicentricity and metastases, especially in familial or malignant cases. On histopathology, CBTs display a zellballen pattern with chromogranin, synaptophysin, and S100 positivity. Malignant potential is suggested by spread to regional lymph nodes or distant metastasis. In our case, histology showed reactive lymph node confirming malignancy. The Shamblin classification remains crucial for surgical planning: Group I - localized, relatively small and minimally attached to carotid vessels; Group II - partially surrounding the vessels with moderate arterial attachment; and Group III - completely encasing the carotid arteries [11]. Our case, being Group III, required meticulous dissection with vascular control and right ICA repair with end to end PTFE graft interposition between right CCA and ICA. Surgical excision of the tumor is the treatment of choice [12]. Preoperative embolization may help in larger lesions to minimize intraoperative bleeding, though its benefit is debated [13]. Radiotherapy is reserved for inoperable, residual, or metastatic disease. Chemotherapy offers limited benefit. Recent literature highlights the importance of genetic screening, particularly of SDHB gene, for early-onset or malignant cases [7-9]. Kimura et al. proposed biochemical and genetic risk stratification frameworks which can help in integrating clinical and molecular features, facilitating individualized surveillance protocols [14]. Our case adds to the limited literature of malignant CBTs in adolescents, reinforcing the importance of early recognition and curative surgical excision.

## Conclusions

Malignant CBTs are exceedingly rare, particularly in young patients. Confirmation of malignancy requires evidence of metastasis because histology alone does not provide a dependable distinction between the benign and malignant forms. This case underscores the importance of maintaining a high index of suspicion when assessing slow-growing lateral neck masses in younger patients. Detailed preoperative imaging, thorough planning, and meticulous surgical technique are crucial for optimal outcomes given the tumor’s close relationship with the major vascular structures. Long-term surveillance is recommended due to the potential for late recurrence even after complete surgical resection. Early diagnosis combined with a multidisciplinary approach can significantly improve the outcomes and can also help guide appropriate decisions regarding surveillance and adjuvant therapy.

## References

[1] Valderrama-Treviño AI, Correa-Posada MO, García-Vélez JF (2024). Carotid body tumors: a review. Int Surg J [Internet.

[2] Boedeker CC (2011). Paragangliomas and paraganglioma syndromes. GMS Curr Top Otorhinolaryngol Head Neck Surg.

[3] Dorobisz K, Dorobisz T, Temporale H (2016). Diagnostic and therapeutic difficulties in carotid body paragangliomas, based on clinical experience and a review of the literature. Adv Clin Exp Med.

[4] Lone GN, Shah AP, Malik PA, Hussain SM, Wani GM (2016). Carotid body tumors: surgical management and review of patients over 10 years. Indian J Vasc Endovasc Surg.

[5] Tabb JN, Maas JA, Earla BP, Fallon KB, McDonald AM, Dobelbower MC (2023). Carotid body paraganglioma metastatic to spine causing cord compression: a case report. Diagn Pathol.

[6] Chen H, Zhu W, Li X, Xue L, Wang Z, Wu H (2017). Genetic and epigenetic patterns in patients with the head-and-neck paragangliomas associate with differential clinical characteristics. J Cancer Res Clin Oncol.

[7] Fishbein L, Khare S, Wubbenhorst B (2015). Whole-exome sequencing identifies somatic ATRX mutations in pheochromocytomas and paragangliomas. Nat Commun.

[8] Calsina B, Currás-Freixes M, Buffet A (2018). Role of MDH2 pathogenic variant in pheochromocytoma and paraganglioma patients. Genet Med.

[9] Majewska A, Budny B, Ziemnicka K, Ruchała M, Wierzbicka M (2020). Head and neck paragangliomas - a genetic overview. Int J Mol Sci.

[10] Pacheco-Ojeda LA (2017). Carotid body tumors: surgical experience in 215 cases. J Craniomaxillofac Surg.

[11] Shamblin WR, ReMine WH, Sheps SG, Harrison EG (1971). Carotid body tumor (chemodectoma): clinicopathologic analysis of ninety cases. Am J Surg.

[12] Wang SJ, Wang MB, Barauskas TM, Calcaterra TC (2000). Surgical management of carotid body tumors. Otolaryngol Head Neck Surg.

[13] Plukker JT, Brongers EP, Vermey A, Krikke A, van den Dungen JJ (2001). Outcome of surgical treatment for carotid body paraganglioma. Br J Surg.

[14] Kimura N, Takekoshi K, Naruse M (2018). Risk stratification on paraganglioma and pheochromocytoma: from laboratory to clinical practice. J Clin Med.

